# Creation and Evaluation of the Illinois Cancer Risk Index as a Predictor of Four Common Cancers

**DOI:** 10.5888/pcd19.220104

**Published:** 2022-11-17

**Authors:** Lei Guo, Margaret E. Wright, Meredith C. Osias, Mahdi Vaezi, M. Courtney Hughes

**Affiliations:** 1 School of Interdisciplinary Health Professions, Northern Illinois University, DeKalb, Illinois; 2 University of Illinois Cancer Center, Chicago, Illinois; 3 School of Health Studies, Northern Illinois University, DeKalb, Illinois; 4 Department of Engineering Technology, Northern Illinois University, DeKalb, Illinois

## Abstract

**Introduction:**

Nearly half of all cancer deaths in the US are attributed to 4 common cancers: lung, colorectal, breast, and prostate. Illinois residents experience higher rates of cancer death from all 4 cancers compared with the US overall. We developed the Illinois Cancer Risk Index (ICRI), which incorporates many predictors of these cancers into a single summary measure, to identify Illinois counties that would benefit most from public health intervention.

**Methods:**

We identified 90 county-level predictors of 4 common cancers, used multicollinearity testing to reduce this number to 61, and applied factor analysis to extract and analyze 4 factors representing 25 variables. Next, we created the ICRI by regressing the 4 factors on our outcome of interest — an age-adjusted common cancers mortality rate (CCMR), incorporating the direction of the β-coefficients from regression models to sum factor scores. Finally, we mapped and assessed the geographic distributions of both ICRI and CCMR by county across the state.

**Results:**

The ICRI was positively associated with the CCMR (*r* = 0.59, *P* < .001) and explained 32.2% of the variance in the CCMR across Illinois. The ICRI showed distinct geospatial patterns across the state, with the highest risk counties located in the east–central, far northern, and southern regions. The CCMR showed similar geospatial patterns.

**Conclusion:**

Our study identifies counties in Illinois that may benefit most from interventions that target multiple cancer risk factors simultaneously. The ICRI may be adapted for use in other geographic locations where data are available.

**Summary**

**What is already known on this topic?**

Information is needed on how social and environmental determinants of health affect outcomes of common cancers to create a measure and identify geographic locations that are most in need of public health intervention.

**What is added by this report?**

A risk index representing 25 predictors of death from the 4 most common cancers was created by using population-based, county-level data in Illinois. We correlated the index with mortality rates from the 4 most common cancers, and both exhibited similar geospatial distribution across the state.

**What are the implications for public health practice?**

Professionals in many health fields can adapt our framework to construct indexes and inform public health resource allocations.

## Introduction

Cancer is the second leading cause of death in the US, with lung cancer accounting for almost one-quarter (23%) of cancer deaths. Other common sites of cancer death are colorectal (9%), female breast (7%), and prostate (5%) (1). Illinois residents experience higher rates of death from all 4 of these cancers compared with the US overall (1). In Illinois, approximately 14,140 deaths from these 4 common cancers are expected in 2022, with wide variation across the state (2).

Determinants of cancer incidence include those related to demographics, social and economic factors, health behaviors, the physical environment, and clinical care (3). For example, Singh et al (4) showed that cancer outcomes in the US correlate with socioeconomic status and race and ethnicity at both individual and population levels. Other researchers have demonstrated that health behaviors and cumulative environmental quality influence cancer risk (5–7). Krieger (8) proposed an ecosocial theory and discussed how social and biologic reasoning and dynamic and ecologic perspectives could affect distributions and result in social inequalities in cancer outcomes. Most cancer risk factors have been examined in isolation with respect to outcomes. Although some indexes have been constructed and used, their data were older (9,10) than ours, focused on just one cancer type (11), or did not include specific risk factors such as individual health behaviors, air pollution, English proficiency, and poor physical and mental health (9–11). Our study included these additional elements of risk and evaluated the index’s relation to an outcome that reflects the 4 most common causes of cancer death in Illinois.

This article introduces the Illinois Cancer Risk Index (ICRI), which incorporates predictors of the most common cancer deaths into a single summary measure. The index can be used to identify counties in Illinois where public health intervention is needed most.

## Methods

We identified 90 variables related to the following domains of risk: demographics, social and economic factors, health behaviors, the physical environment, and clinical care. We selected these on the basis of published literature (3), ecosocial theory (8), and available Illinois county data. For each variable, the most recent county-level data (2014–2018) were extracted from publicly available sources, including the Area Health Resources Files (12), County Health Rankings and Roadmaps (CHR) (13), the US Department of Agriculture Food Environmental Atlas (14), and the Illinois Environmental Protection Agency Bureau of Air (15).

We normalized each of the 90 variables and expressed them as either per-capita values or percentages by using the following formula:

Variable _normalized_ = (Variable – Variable _minimal_) / (Variable _maximal_ – Variable _minimal_)

Data were available for more than 94% of our study variables for all 102 Illinois counties. We replaced missing data for the risk factors (1.1% of data points missing) and site-specific cancer mortality rates (30.4% of data points missing) by using the hot deck imputation method (16). Subsequently, we standardized data for each variable by applying a *z* score standardization to transform the different variables into comparable scales (16). We then conducted redundancy and multicollinearity tests by using variance inflation factors (VIFs) to remove highly correlated variables (VIF >5) and improve the efficiency of factor analysis. Sixty-one variables were retained for subsequent exploratory factor analysis (16).

Exploratory factor analysis was used to extract 13 grouped variables called “factors” from these 61 variables. Analysis was initiated by estimating the variance component with principal component analysis. We used the Kaiser Measure of Sampling Adequacy (Kaiser MSA) in combination with variable communalities (proportion of each variable’s variance that the factors explain) to extract these factors. We used the Bartlett χ^2^ test to validate the estimated factors (all *P *values < .001). Factors containing 2 or fewer variables or those whose ascribed variables all had factor loadings less than 0.5 were excluded. Four factors representing 25 variables were retained for analysis (Table 1).

**Table 1:** Factor Loadings^a^ and Sources of Data for Variables, the Illinois Cancer Risk Index, 2014–2018

Variable	Factor name^b^					
	1. Black race and health behaviors	3. Ethnicity, air quality, housing, and rurality	5. Financial needs and unemployment	6. Education, primary care provider, and income ratio	Communalities^c^	Source
Non-Hispanic Black population, 2014–2018, %	0.93^d^	—	—	—	0.96	County Health Rankings (13)
Non-Hispanic White population, 2014–2018, %^b^	—	−0.79	—	—	0.95	
Hispanic population, 2014–2018, %	—	0.79	—	—	0.90	
Rural population, 2010, %	—	0.75	—	—	0.81	
Current smoker^d^, %	0.88	—	—	—	0.94	
Excessive drinking^e^, %	0.85	—	—	—	0.90	
Poor physical health days^f^, mean no.	0.79	—	—	—	0.85	
Poor mental health days^g^, mean no.	0.62	—	—	—	0.89	
Limited access to healthy foods, 2014–2018, %	0.53	—	—	—	0.86	
Severe housing problems^h^, %	—	0.73	—	—	0.86	
Carbon monoxide^i^	—	0.72	—	—	0.97	Illinois Environmental Protection Agency Bureau of Air (15)
Nitrogen oxides^j^	—	0.72	—	—	0.97	
Average daily PM_2.5_ k	—	0.72	—	—	0.65	County Health Rankings (13)
Sulfur dioxide^l^	—	0.72	—	—	0.97	Illinois Environmental Protection Agency Bureau of Air (15)
Volatile organic material^m^	—	0.72	—	—	0.97	
Population not proficient in English, %	—	0.69	—	—	0.90	County Health Rankings (13)
Population living in poverty, 2014–2018, %	—	—	0.82	—	0.93	Area Health Resources Files (12)
Median household income, 2014–2018	—	—	−0.81	—	0.95	County Health Rankings (13)
Median home value, 2014–2018	—	—	−0.66	—	0.94	Area Health Resources Files (12)
Median gross rent, 2014–2018	—	—	−0.60	—	0.89	
Unemployment rate, 2014–2018	—	—	0.56	—	0.72	USDA Food Environmental Atlas (14)
Some college^n^	—	—	—	−0.51	0.78	County Health Rankings (13)
Ratio of population to primary care physicians	—	—	—	0.55	0.7	
Income ratio^o^	—	—	—	0.54	0.743	
Correlation coeffect *r* and *P* value	*r* = 0.37, *P* < .001	*r* = 0.36, *P* < .001	*r* = 0.04, *P* = 0.03	*r* = 0.26, *P* < .001	—	—

Abbreviations: — , variable not included in the model.

a Factor loadings: correlation coefficients between observed variables and common latent factors.

b Factor is a latent variable associated with a set of observed variables that have similar response patterns.

c The correlation coeffect *r* and *P* value of bivariate linear regression between factor and common cancers mortality rate.

d Percentage of adults who smoked 100 cigarettes in their lifetime and currently smoked, 2014–2018.

e Percentage of adults reporting binge or heavy drinking, 2014–2018. Binge drinking = consuming 4 or more drinks on one occasion for a woman or 5 or more drinks on one occasion for a man. Heavy drinking = 8 or more drinks per week for a woman or 15 or more drinks per week for a man.

f Number of physically unhealthy days in past 30 days (age-adjusted), 2014–2018.

g Number of mentally unhealthy days in past 30 days (age-adjusted), 2014–2018.

h Percentage of households with at least 1 of 4 housing problems: overcrowding, high housing costs, lack of kitchen, or lack of plumbing facilities, 2014–2018.

i Carbon monoxide stationary point source emission distribution at county level (tons/y), 2014–2018.

j Nitrogen oxides stationary point source emission distribution at county level (tons/y), 2014–2018.

k Average daily density of fine particulate matter in micrograms per cubic meter (PM_2.5_), 2014–2018.

l Sulfur dioxide stationary point source emission distribution (tons/y), 2014–2018.

m Volatile organic material point source emission distribution (tons/y), 2014–2018.

n Adults aged 25–44 years with some post-secondary education, 2014–2018.

o Ratio of household income at the 80th percentile to income at the 20th percentile at county level, 2014–2018.

Our outcome of interest was the average age-adjusted mortality rate from lung, colorectal, breast, and prostate cancers, which we refer to as the common cancer mortality rate (CCMR). Cancer mortality data were obtained from the Illinois State Cancer Registry and included the years 2014–2018 (15). We replaced missing data with hot-deck imputation for counties that had low counts because of suppressed data.

We used multiple linear regression to assess the bivariate and multivariate relationships between each of the 4 retained factors. We then used this regression analysis information to construct the ICRI. In brief, the direction of each association (the sign of the β-coefficient) was incorporated into the calculation of the ICRI. For each Illinois county, the ICRI was calculated by multiplying the standardized value of each variable by its respective factor loading and then summing all 4 factors together while accounting for the sign of the β-coefficient from regression models. We used the following equation:

ICRI = Factor Score 1 + Factor Score 3 + Factor Score 5 + Factor Score 6

For example, we calculated the ICRI for Cumberland County as

ICRI_Cumberland County_ = Factor Score 1_Cumberland County_ + Factor Score 3_Cumberland County_ + Factor Score 5_Cumberland County_ + Factor Score 6_Cumberland County =_ (−0.49) + (−1.91) + (−3.92) + (−3.05) = −9.37

Sensitivity analyses were performed in which the ICRI was regressed individually against breast, colorectal, lung, and prostate cancer mortality rates.

We generated and comparted maps of the ICRI and CCMR by county. We divided county-level CCMRs into quintiles for mapping. For the ICRI map, counties were classified for risk as very low, low, average, high, and very high. Low- and high-risk counties had index values that were more than 0.5 SD but less than 1.5 standard deviations from the mean. Very low-risk and very high-risk counties had an index more than 1.5 standard deviations from the mean. Average risk counties had an ICRI value within 0.5 standard deviations from the mean.

We used SPSS version 27.0 (IBM Corp) to conduct all analyses and constructed maps in ArcGIS Pro 2.8 (Esri Corp).

## Results

The communalities of variables (the proportion of common variance found in a particular variable) were all higher than 0.5. All variables correlated with at least 1 other variable (correlation coefficient of at least 0.3), indicating that variables all shared some common variance. We retained 4 factors representing 25 variables because, together, they explained 70.1% of the total variance of the 61 originally identified, noncollinear variables across Illinois. These factors were calculated, along with the variables that each one comprises and their factor loadings (Table 1). Factor 1 included Black race and health behaviors (the variable with the highest factor loading was Black race at 0.931) and explained 27.3% of the total variance. Factor 3 included Hispanic ethnicity, rurality, measures of air pollution, and language barriers (the variable with the highest factor loading was the percentage of non-Hispanic White race at −0.787) and explained 17.8% of the total variance. Factor 5 included aspects of financial security (the variable with the highest factor loading was percentage of people living in poverty at 0.820) and explained 16.7% of the total variance. Factor 6 included density of primary care providers, education, and income ratio (the variable with the highest factor loading was the primary care provider [PCP] rate at 0.553). PCP rate is the ratio of population to primary care physicians and includes practicing physicians specializing in general practice medicine, family medicine, internal medicine, and pediatrics, and explained 8.3% of the total variance.

### Regression analysis and index construction

In bivariate regression, Factor 5 was the only significant predictor of the CCMR; Factors 1, 3, and 6 and the ICRI were not. However, in multivariable regression, all 4 retained factors significantly predicted the CCMR. Together, they explained 33.5% of the variance in the county-level CCMR across the state of Illinois (Table 2).

**Table 2:** Associations Between Retained Factors and the Illinois Cancer Risk Index (ICRI) with the Common Cancer Mortality Rate, 2014–2018

Factor name	Multivariable linear regression^a^			Bivariate linear regressions				
	β	SE	*P* value	Intercept	β	SE	*P* value	*r* ^2^
**Intercept**	20.65	0.02	<.001	—	—	—	—	—
**Factor 1**	0.23	0.08	<.001	20.64	0.76	0.09	<.001	0.14
**Factor 3**	2.11	0.07	<.001	20.65	1.49	0.08	<.001	0.13
**Factor 5**	0.04	0.08	.01	20.64	0.19	0.09	.03	0.002
**Factor 6**	0.38	0.11	.004	20.66	0.65	0.09	<.001	0.07
**ICRI**	—	—	—	20.63	1.09	0.16	<.001	0.32

Abbreviations: — , variable not included in the model.

a *r^2^* = 0.335.

The ICRI had a moderate positive association with the CCMR (*r* = 0.59, *P* < .001) and explained 32.2% of the variance in this outcome (Table 2). The ICRI had a mean of 0 and an SD of 4.6. Winnebago County in the northern portion of the state had the highest index value at 10.76, implying a high risk of mortality from lung, breast, prostate, and colorectal cancers in this area. Cumberland County in the east had the lowest value at −9.37, implying a low risk of mortality from these 4 cancers in that area.

We performed sensitivity analyses by regressing the ICRI against individual cancer mortality rates rather than the CCMR (Table 3). The ICRI explained the largest proportion of variation in prostate cancer mortality (64%), followed by deaths from breast (57%), colorectal (56%), and lung (23%) cancer.

**Table 3:** Regression Analysis, Illinois Cancer Risk Index (ICRI) Relative to Breast, Colorectal, Lung, and Prostate Cancer Mortality Rates, 2014–2018

Age-adjusted cancer mortality rates (2014–2018)	Bivariate linear regressions				
	Intercept	β	SE	*P* value	*r* ^2^
Breast cancer	12.483	1.37	0.20	<.001	0.57
Colorectal cancer	12.324	0.10	0.13	<.001	0.56
Lung cancer	51.500	0.39	0.25	.02	0.23
Prostate cancer	8.116	1.33	0.16	<.001	0.64

### Maps

Thirty-three Illinois counties were classified as at high or very high risk for the 4 common cancers (Figure) based on the ICRI, and 32 counties were classified as low or very low risk. Higher risk counties were primarily located in the east–central portion of the state, with several counties in the far northern and southern portions of the state also classified as such. Counties in the 2 highest CCMR quintiles were located predominantly in the northeast and southern parts of Illinois, with some counties in the central portion.

**Figure 1:**
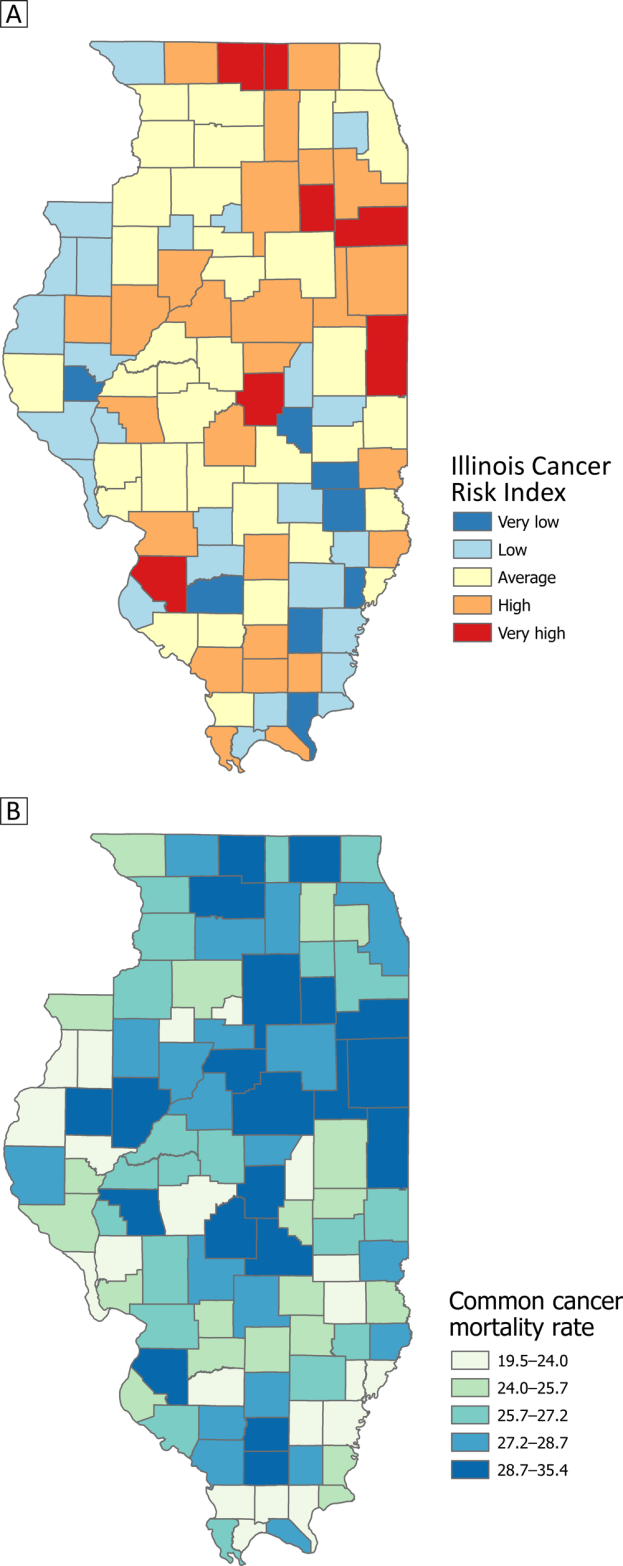
Distribution by county of risk of the 4 most common cancers — lung, colorectal, breast, and prostate — in Illinois. Map A displays risk by the Illinois Cancer Risk Index (ICRI) for each county. Higher risk counties were located in the east-central portion of the state, with some also located in the far northern and southern portions of the state. Map B plots the common cancer mortality rate (CCMR), 2014–2018, for each county in Illinois. Counties in the 2 highest CCMR quintiles were located in the northeast and southern parts of Illinois, with some also located in the central portion of the state.

**Table:** 

County	ICRI	Levels, Illinois Cancer Risk Index	CCMR range
Adams	2.216799338	Average	27.2–28.7
Alexander	2.444087695	High	25.7–27.2
Bond	–4.380578947	Low	25.7–27.2
Boone	7.215588251	Very high	25.7–27.2
Brown	–7.298549372	Very low	24.0–25.7
Bureau	–1.43071986	Average	24.0–25.7
Calhoun	–4.855307172	Low	19.5–24.0
Carroll	–2.176511892	Average	25.7–27.2
Cass	0.86555653	Average	25.7–27.2
Champaign	–0.445723063	Average	24.0–25.7
Christian	5.466752813	High	28.7–35.4
Clark	3.496914011	High	27.2–28.7
Clay	1.98102252	Average	24.0–25.7
Clinton	–3.984181667	Low	24.0–25.7
Coles	–0.041119708	Average	25.7–27.2
Cook	0.990786352	Average	27.2–28.7
Crawford	–2.287715925	Average	24.0–25.7
Cumberland	–9.36780711	Very low	19.5–24.0
DeKalb	3.716485961	High	27.2–28.7
DeWitt	2.65950246	High	27.2–28.7
Douglas	–6.484072014	Low	24.0–25.7
DuPage	–2.539425674	Low	24.0–25.7
Edgar	0.891185364	Average	25.7–27.2
Edwards	–7.668748421	Very low	19.5–24.0
Effingham	–5.379371494	Low	24.0–25.7
Fayette	0.68872117	Average	27.2–28.7
Ford	2.737998405	High	28.7–35.4
Franklin	4.720887939	High	28.7–35.4
Fulton	4.481021702	High	28.7–35.4
Gallatin	–5.212315704	Low	24.0–25.7
Greene	–1.754580215	Average	19.5–24.0
Grundy	8.882054859	Very high	28.7–35.4
Hamilton	–7.087406251	Very low	19.5–24.0
Hancock	–4.256978672	Low	19.5–24.0
Hardin	–5.582425961	Low	24.0–25.7
Henderson	–5.121135896	Low	19.5–24.0
Henry	0.513533619	Average	25.7–27.2
Iroquois	2.601617754	High	28.7–35.4
Jackson	2.507206441	High	27.2–28.7
Jasper	–8.401371317	Very low	19.5–24.0
Jefferson	1.770525053	Average	27.2–28.7
Jersey	–1.258764951	Average	24.0–25.7
Jo Daviess	–5.30041805	Low	24.0–25.7
Johnson	–5.195538373	Low	19.5–24.0
Kane	1.841744962	Average	24.0–25.7
Kankakee	9.379546338	Very high	28.7–35.4
Kendall	4.681568249	High	25.7–27.2
Knox	1.332599524	Average	27.2–28.7
Lake	0.870935351	Average	28.7–35.4
LaSalle	5.669163712	High	24.0–25.7
Lawrence	2.48515083	High	27.2–28.7
Lee	0.706769345	Average	27.2–28.7
Livingston	1.500310072	Average	25.7–27.2
Logan	0.260141218	Average	25.7–27.2
Macon	3.308056677	High	27.2–28.7
Macoupin	5.608384825	High	28.7–35.4
Madison	2.604629481	High	28.7–35.4
Marion	8.120485529	Very high	28.7–35.4
Marshall	–0.185686442	Average	25.7–27.2
Mason	5.242558895	High	25.7–27.2
Massac	2.671288898	High	24.0–25.7
McDonough	–0.097517126	Average	27.2–28.7
McHenry	1.552356659	Average	25.7–27.2
McLean	3.141181222	High	25.7–27.2
Menard	–0.119184555	Average	25.7–27.2
Mercer	–4.06600076	Low	24.0–25.7
Monroe	–2.870841123	Low	24.0–25.7
Montgomery	1.960420187	Average	27.2–28.7
Morgan	2.837240829	High	28.7–35.4
Moultrie	–8.621253698	Very low	24.0–25.7
Ogle	1.849274423	Average	27.2–28.7
Peoria	6.099818927	High	27.2–28.7
Perry	1.199605416	Average	27.2–28.7
Piatt	–6.257683417	Low	19.5–24.0
Pike	–2.874877121	Low	24.0–25.7
Pope	–6.992855878	Very Low	19.5–24.0
Pulaski	–5.067809674	Low	19.5–24.0
Putnam	–4.89849348	Low	19.5–24.0
Randolph	–0.780709522	Average	25.7–27.2
Richland	–2.505624738	Average	24.0–25.7
Rock Island	1.138232555	Average	25.7–27.2
Saline	9.045043492	Very high	28.7–35.4
Sangamon	2.554330748	High	27.2–28.7
Schuyler	0.414808163	Average	19.5–24.0
Scott	–4.397325493	Low	19.5–24.0
Shelby	–3.149582297	Low	25.7–27.2
St. Clair	–1.054286324	Average	28.7–35.4
Stark	–5.81489276	Low	19.5–24.0
Stephenson	4.498989772	High	27.2–28.7
Tazewell	3.266639608	High	27.2–28.7
Union	–0.583847446	Average	19.5–24.0
Vermilion	10.15660931	Very high	28.7–35.4
Wabash	–1.689835769	Average	19.5–24.0
Warren	–4.303920216	Low	19.5–24.0
Washington	–7.12420722	Very low	19.5–24.0
Wayne	–3.096825135	Low	25.7–27.2
White	–6.707720343	Low	19.5–24.0
Whiteside	0.248622401	Average	25.7–27.2
Will	6.167540737	High	25.7–27.2
Williamson	5.760631093	High	28.7–35.4
Winnebago	10.77589956	Very high	28.7–35.4
Woodford	0.972921003	Average	27.2–28.7

## Discussion

We constructed a novel cancer risk index — ICRI — by using population-based, county-level data from the state of Illinois. The ICRI represented a broad range of determinants of the 4 most common cancers in both Illinois and the US. To the best of our knowledge, our study incorporates one of the largest numbers of cancer risk factors (based on ecosocial theory [8]) to date and has important implications for screening, intervention resource allocation, and access to cancer care.

Our study differs from other reports that also describe cancer risk indexes. Although Scott et al (9) included factors from several domains, our study also examined air pollution, English proficiency, and poor physical and mental health. Wang et al (10) used data from 1998–2000 to focus exclusively on late-stage cancers and did not include rurality, air pollution, English proficiency, poor physical and mental health, or health behaviors such as alcohol consumption, smoking, and diet. In a separate publication, Wang et al (11) used breast cancer mortality throughout Illinois as the outcome of interest and did not include rurality, air pollution, English proficiency, poor physical and mental health, health behaviors, or ratio of population to primary care physicians. Overall, our study evaluated more domains than previous studies and created a unique outcome by averaging the 4 most common causes of cancer death in Illinois.

Demographic variables had high loadings in 2 factors included in our index. For the first factor, the percentage of the population that identified as non-Hispanic Black had the highest factor loading. For the second factor, the percentage of the population that identified as non-Hispanic White had the highest factor loading. Together, these 2 factors explained 45.1% of the total variance of the 61 variables across Illinois. These variables exhibited the connections between social disparities and cancer risks across Illinois counties, showing patterns similar to larger-scale studies. For instance, previously published research found substantially elevated cancer mortality rates at multiple sites among non-Hispanic Black populations compared with non-Hispanic White populations (8). On the other hand, Hispanic populations had lower cancer mortality rates than non-Hispanic White or non-Hispanic Black populations, although Hispanic populations tend to have later-stage diagnoses and poorer quality of life at the national level and across Illinois (1).

In our index, Hispanic ethnicity is loaded passively with air pollution variables. This association may indicate the disparities between racial and ethnic minorities and environmental pollution (7). A recent study showed that Hispanic, Latino, and other minority populations were being exposed to higher levels of dangerous fine particulate matter 2.5 (PM_2.5_) from air pollution than other groups; previous studies also found that the Hispanic population is at higher risk of premature death from exposure to PM_2.5 _air pollution (17), which echo our results. Next, because about 14% of the Illinois population are non–US-born (18), it is important to consider the additional hurdles encountered by this group. Limited English proficiency can burden non–US-born people when they attempt to access health care in the US, although they often have lower cancer mortality rates than people born in the US. Over time, their cancer rates tend to equal or even exceed those of people born in the US as they acculturate, and this tendency is why English proficiency was loaded in our index.

Factor 1 included health behavior–related variables and poor physical and mental health days. Multiple studies have shown that health behaviors are strongly associated with cancer outcomes. For example, smoking and alcohol consumption are known risk factors for multiple cancers (19,20), with smoking the most predominant risk factor for lung cancer (3). Additionally, previous research demonstrated that people who develop mental disorders after a cancer diagnosis may be at higher risk of cancer death (21). Furthermore, mental health treatment offered to cancer patients after diagnosis can improve lung cancer survival, and reductions in the severity of mental illness may manifest in greater self-efficacy for managing chronic conditions and improvements in positive health behaviors, such as physical activity and stress management (22). However, past studies using Illinois data (10,11) and US data (9) did not consider these variables to create their single or multiple cancer sites index.

Living in a rural area — which had a high factor loading in our index — is also associated with higher cancer mortality rates because of limited health care access (23). Another variable, severe housing problems, was loaded in the same factor with rural area. Although housing is not frequently examined with cancer outcomes, studies have found that it is associated with increased cancer mortality disparities between Black and White populations (9,23). Rural residency and the health behavior variables loaded in our factors might represent latent variables related to protective behaviors associated with cancer outcomes.

Household income, median home value, and median gross rent were loaded negatively in Factor 5, echoing the findings from other research that low- and middle-income counties have higher cancer mortality rates than high-income counties in the US (24). On the other hand, the variables “some college,” “PCP rate,” and “income ratio” were positively associated in Factor 6. These results are similar to some studies that found that education, primary care access, and income might have essential roles in cancer treatment and prevention (25,26). For example, use of breast and colorectal cancer screening is 20% to 30% lower among those with only a high school education than among college graduates (25). Also, a greater primary care physician supply was associated with lower cancer mortality (26), and low income is a barrier to health care access (25).

Although the ICRI explained only 32.2% of the variance in the age-adjusted CCMR, the combinations of the multiple cancer risk variables demonstrate the nature of cancer as a heterogeneous disease with many risk factors that may have a long-term impact on health (8). Although resource allocation can affect all the variables analyzed in this article, some variables, such as smoking and alcohol consumption, are also considered modifiable cancer risk factors. In addition, lifestyle modification could significantly reduce the burden of cancer (27); thus, public health professionals in Illinois may use the index to direct risk reduction and health promotion programs and policies at those counties most in need.

Our index explained the largest proportion of variance in prostate cancer mortality rates across Illinois and the smallest proportion for lung cancer. Many variables used in our study may relate to prostate cancer mortality in Illinois compared with other cancer types because early screening often can reduce prostate cancer risk for highly educated people with cancer screening resources (28). The low prediction of lung cancer mortality might be due to the noise introduced by smoking as the strongest risk factor for lung cancer (3). It also indicates that underlying latent sociodemographic variables other than smoking and air pollution could have an impact on lung cancer mortality in Illinois. Future studies could explore more variables specifically related to lung cancer mortality risk. Nevertheless, our index map showed a similar geospatial pattern that matches the county-level CCMR. Both maps demonstrate the compelling need for cancer-related public health resource allocation in east-central and northern Illinois.

Previous Illinois studies examined the associations between several risk factors and late-stage diagnosis for 4 common cancers and created a county-level index for breast cancer (10,11). Our study differs in that we comprehensively focused on ecosocial determinants of cancer risk factors according to ecosocial theory (8). Many variables used to construct our index are also associated with other cancer outcomes and other chronic noncommunicable diseases (27,29). This framework could potentially be used to create a similar scoring system for public health professionals.

### Strength and limitations

Our study had several strengths. First, it incorporated numerous cancer risk factors at population levels to create a cancer risk index for Illinois. Our results identified physical and mental health variables and air toxin variables that previous studies did not include. Additionally, the use of a factor analysis framework can explore the underlying trends in the data, increase interpretability, and minimize information loss while reducing dimensionality. Furthermore, we identified and reduced factors from large feature sets associated with common cancer mortality in Illinois, and this information can assist in cancer intervention and prevention program planning at the county level.

Our study had some limitations. First, we only examined the state of Illinois. Further studies should explore larger data sets with counties from multiple US states and abroad. Second, we needed to impute data for some counties because of missing data; this was to ensure that an ICRI value was ascribed to each county. Third, we relied on data that were self-reported and aggregated at the county level, which may obscure nuances in individual behavior and be susceptible to social desirability bias. Fourth, caution must be applied in interpreting the index because we evaluated the ICRI by using its correlation with CCMR, and correlation does not signify causation. Despite these limitations, the calculated index provides informative data to advise public health professionals. To address these limitations in future studies, researchers can use larger and validated data sets and machine learning frameworks, which are becoming increasingly prevalent in cancer research, to model risk factors (30).

### Conclusion

This study identified, reduced, and analyzed a substantial number of cancer predictors and incorporated them into a single novel county-level index for the state of Illinois. Our analysis found that the ICRI was moderately associated with the CCMR, which is the average mortality from the 4 most common cancers in the state. Public health professionals may use this framework to target resources and interventions to counties in Illinois that score highest on the risk index and are, therefore, most in need. Future research should apply this framework to construct indexes for other diseases and for multiple geographic locations.
